# Gastrointestinal malignancies harbor actionable MET exon 14 deletions

**DOI:** 10.18632/oncotarget.4721

**Published:** 2015-09-10

**Authors:** Jeeyun Lee, Sai-Hong Ignatius Ou, Ji Min Lee, Hee Cheol Kim, Mineui Hong, Sun Young Kim, Jiryeon Jang, Soomin Ahn, So Young Kang, Sujin Lee, Seung Tae Kim, Bogyou Kim, Jaehyun Choi, Kyung-Ah Kim, Jiyun Lee, Charny Park, Se Hoon Park, Joon Oh Park, Ho Yeong Lim, Won Ki Kang, Keunchil Park, Young Suk Park, Kyoung-Mee Kim

**Affiliations:** ^1^ Department of Medicine, Division of Hematology-Oncology, Samsung Medical Center, Sungkyunkwan University School of Medicine, Seoul, Korea; ^2^ Innovative Cancer Medicine Institute, Samsung Medical Center, Seoul, Republic of Korea; ^3^ Chao Family Comprehensive Cancer Center, University of California Irvine School of Medicine, Orange, California, USA; ^4^ Samsung Biomedical Research Institute, Samsung Advanced Institute of Technology (SAIT)/Samsung Electronics Co. Ltd, Yeongtong-gu, Suwon-si, Gyeonggi-do, Korea; ^5^ Department of Surgery, Samsung Medical Center, Sungkyunkwan University School of Medicine, Seoul, Korea; ^6^ Department of Pathology and Translational Genomics, Samsung Medical Center, Sungkyunkwan University School of Medicine, Seoul, Korea

**Keywords:** MET exon 14 skipping, colorectal carcinoma, MET monoclonal antibodies, crizotinib, gastrointestinal malignancies

## Abstract

Recently, *MET* exon 14 deletion (*METex14del*) has been postulated to be one potential mechanism for MET protein overexpression. We screened for the presence of *METex14del* transcript by multiplexed fusion transcript analysis using nCounter assay followed by confirmation with quantitative reverse transcription PCR with correlation to MET protein expression by immunohistochemistry (IHC) and *MET* amplification by fluorescence *in situ* hybridization (FISH). We extracted RNAs from 230 patients enrolled onto the prospective molecular profiling clinical trial (NEXT-1) (NCT02141152) between November 2013 and August 2014. Thirteen *METex14del* cases were identified including 3 gastric cancer, 4 colon cancer, 5 non-small cell lung cancer, and one adenocarcinoma of unknown primary. Of these 13 *METex14del* cases, 11 were MET IHC 3+ and 2 were 2+. Only one out of the 13 *METex14del* cases was *MET* amplified (MET/CEP ratio > 2.0). Growths of two (gastric, colon) *METex14del*+ patient tumor derived cell lines were profoundly inhibited by both MET tyrosine kinase inhibitors and a monoclonal antibody targeting MET. In conclusion, *METex14del* is a unique molecular aberration present in gastrointestinal (GI) malignancies corresponding with overexpression of MET protein but rarely with *MET* amplification. Substantial growth inhibition of *METex14del*+ patient tumor derived cell lines by several MET targeting drugs strongly suggests *METex14del* is a potential actionable driver mutation in GI malignancies.

## INTRODUCTION

Aberrations in the hepatocyte growth factor (HGF)-mesenchymal-epithelial transition (MET) receptor tyrosine kinase axis are frequent in solid malignancies [1]. One such aberration is the overexpression of the MET protein as determined by immunohistochemistry (IHC) which may be associated with MET amplification. MET amplification is present in about 2.6% among 1,115 patient tumors assayed [2]. For example, the frequency of *MET* amplification is rare in gastroesophageal/gastric cancer [3] while MET protein overexpression has been reported in higher incidence [4]. The discordance between low *MET* amplification and high MET protein expression indicates there are other potential mechanisms that can lead to MET overexpression. One such mechanism is *MET* exon14 deletion (*METex14del)* where part of the transmembrane portion and region for the Casitas B-lineage lymphoma (Cbl) E3 ligase-mediated degradation is deleted leading to delay degradation of MET and hence its overexpression (Supplementary Figure S1) [5, 6].

*METex14del* was initially described in 2006 in non-small cell lung cancer (NSCLC) and was caused by mutation in the splice donor site in intron 14 and intronic sequence deletions around *MET* exon 14 [5]. The presence of *METex14del* in NSCLC has subsequently been confirmed by RNA sequencing and whole genome sequencing [7, 8]. Additionally, *METex14del* has been reported in gastric cancer (GC) cell line Hs746T [9, 10] and neuroblastoma [11] indicating this is a potential common mechanism for a variety of tumors to delay the ubiquitination and down-regulation of MET protein leading to its overexpression [5].

We investigated patients with metastatic solid malignancies primarily gastrointestinal (GI) and lung malignancies for the presence of *METex14del* using multiplexed fusion transcript detection assay and then confirmed with reverse transcription PCR (RT-PCR) correlated the MET protein expression and *MET* amplification in *METex14del+* cases. We further generated patient derived tumor cell lines and screened them for the presence of *METex14del* and investigated the consequence of MET inhibition in these *METex14del+* cells lines.

## RESULTS

**Figure 1:**
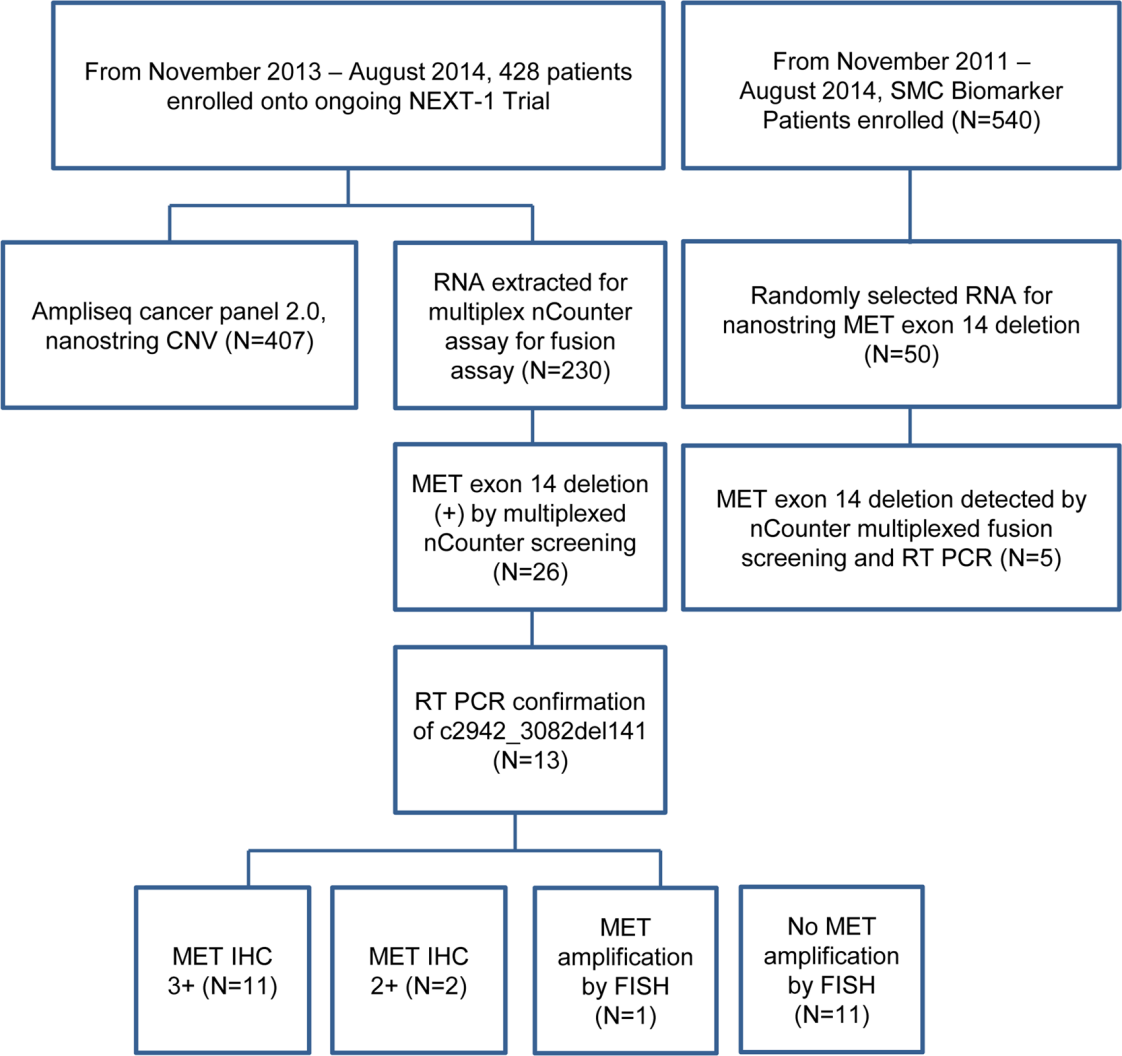
Study flow chart

The patient cohort from the NEXT-1 trial (NCT02141152), which is an actively enrolling clinical trial for genomic profiling in cancer patients, was used (Figure 1). Of 428 patients enrolled and screened, sufficient RNAs for multiplexed fusion transcript detection analysis by nCounter assay were available in 230 patients (Table 1). The detailed probe design for multiplexed fusion transcript assay surveying for ALK, ROS1, RET, NTRK1, and NTRK3 is provided in Supplementary Table S1. Of the multiplexed fusion assay, a nanostring probe to detect any 141bp *METex14del* transcript (p.982_1028del47, c.2942 (Supplementary Table S1) was included. Of the 230 tumor specimens screened, 86 specimens were freshly frozen tissues and 144 specimens were from formalin-fixed paraffin-embedded (FFPE) tissues. In parallel, we screened fifty patient derived tumor cell (PDC) lines generated from the SMC Biomarker study (NCT01831609) for *METex14del*. The SMC Oncology Biomarker study is an ongoing study which enrolls metastatic solid cancer patients with malignant ascites, malignant pleural effusion, endoscopic biopsies or surgical specimens for PDC model establishments (Figure 1 for Study Flow Chart).

**Table 1:** Patient characteristics

Variable	N (%)	MET exon 14 deletion (+)
**Patient tumor specimens (*N* = 230)**		
**Age-year**		
Median	57	
Range	20–87	
**Sex, no. (%)**		
Male	134	
Female	96	
**Stage**	230 (100)	
Tumor type, no. (%)***		
Gastric cancer	42	3(7.1)
NSCLC	51	5(9.8)
Colon cancer	43	4(9.3)
Rectal cancer	23	0(0.0)
Hepatocellular carcinoma	15	0(0.0)
Sarcoma	9	0(0.0)
Pancreatic cancer	5	0(0.0)
Cholangiocarcinoma	6	0(0.0)
Melanoma	5	0(0.0)
ACUP*	3	1(33.3)
Esophageal squamous carcinoma	1	0(0.0)
Renal cell carcinoma	1	0(0.0)
Others	15	0(0.0)
**Patient Derived Cells (*N* = 50)**		
**Patient derived cells (*N* = 50)**		
Gastric cancer	22	1
Colon cancer	5	1
NSCLC	4	1
Melanoma	2	1
Cholangiocarcioma	3	0
HCC**	4	0
Duodenal carcinoma	1	0
Esophageal squamous cell	1	1
Sarcoma and other rare cancer	8	0

* (Adenocarcinoma of unknown primary had met exon 14 skipping and MET amplification).

** HCC, hepatocellular carcinoma.

*** 219 included for final analysis from 230.

Of the 230 tumor cohort (86 fresh frozen tissue and 144 formalin-fixed paraffin-embedded tissues), 219 were finally included in the analysis as 11 samples failed to pass QC (quality control). With initial screening of multiplexed nCounter fusion transcript analysis, 26 were detected as potential positive cases for *METex14del* with high fusion transcript mRNA expression (Supplementary Figure S2) and 13 (5.7%) patients were eventually confirmed to be *METex14del*+ by quantitative reverse transcriptase-polymerase chain reaction (RT-PCR): 3 gastric carcinoma (GC), 4 colon, 5 non-small cell lung cancer (NSCLC) and one adenocarcinoma of unknown primary (ACUP). Among these 13 *METex14del* cases, 11 cases were MET IHC 3+ and 2 cases were MET IHC 2+. Only one of the 13 *METex14del*+ cases had concomitant *MET* amplifications (Table 2). All *METex14del* cases were negative for ALK, ROS1, RET, NTRK1, and NTRK3 fusion.

**Table 2:** Characteristics of MET exon 14 deletion (*METex14*) patients according to tumor types

	Colon	Gastric	ACUP	Lung
**N**	4	3	1	5
**Age**				
**Median**	63	53		49
**(range)**	42–87	27–67		36–60
**Gender**				
**Male**	2	2	1	1
**Female**	2	1	0	4
**Smoking history**				
**Never-smoker**	NC	NC	NC	4
**Ever-smoker**				1
**Histology**				
**Adenocarcinoma**	4	3	1	4
**Squamous cell**	0	0	0	1
**Large cell neuroendocrine**	0	0	0	0
**Undifferentiated**	0	0	0	0
**Tumor differentiation**				
**Well**	1	0	0	0
**Moderate**	2	0	0	1
**Poor**	1	3	1	4
**MET IHC**				
**0**	0	0	0	0
**1+**	0	0	0	0
**2+**	1	0	0	0
**3+**	3	3	1	5
**Concomitant MET amplification**				
**Yes**	0	1	0	0
**No**	4	2	0	5
**Confirmed by RT-PCR**				
**Yes**	4	3	1	5
**No**	0	0	0	0

NC, not contributable; wt, wild type.

All 13 *METex14del* cases were further confirmed by qualitative RT-PCR using probes overlapping an exon 13–15 junction, a fusion transcript caused by exon 14 skipping. In all cases, although the absolute Ct (cycles to threshold) values of RT-PCR showed relatively high around 32, there was definite amplification of target sequences. Deep sequencing targeting whole *MET* gene including intron using DNAs from GI cancers, there were many mutations in the introns (Table 3). Interestingly, all our GI samples harbored c.3082+811A TTTTAACA > GGTTTGAT mutations on intron 14 region of *MET*.

**Table 3:** Clinicopathologic characteristics of the gastrointestinal cancer and adenocarcinoma of unknown primary with *METex14del*

Patient Number	Gender	Age	Site of tumor	Clinical findings	MET IHC	Nanostring	MET amplification (CEP7/MET)	Genetic alterations	MET variants detected in deep sequencing
PS-14-482	F	61	Descending colon	Colon cancer with peritoneal seeding	2+	173.42	No (1:1)	KRAS-wild BRAF V600E mutation Microsatellite-stable	c.3082+811TTTTAACA > GGTTTGAT (0.189) c.3082+115ATTTACCTC > TTGTTTGTT (0.095) c.3082+980CTATT > GATAA (0.121) c.3083–730del1 (0.058) c.3083–719TT > GA (0.056) c.3083–257AAGCA > GATCT (0.053)
PS-14-491	M	42	Sigmoid colon	Colon cancer with multiple lymph node metastasis	3+	174.29	No (1:1.2)	KRAS-wild BRAF-wild TP53 and CTNNB1 mutation Microsatellite-unstable	c.3082+811TTTTAACA > GGTTTGAT (0.140) c.2941+24T > C (0.262) c.3082+388TTGA > AATT (0.055) c.3082+716T > C (0.382) c.3083–1164TTTT > AGAC (0.057) c.3083–730del1 (0.061) c.3083–718TCTCC > CAGTT (0.071)
PS-14-536	M	87	Descending colon	Colon cancer with obstruction, seeding	3+	51.61	No (1:0.9)	KRAS-wild BRAF-wild TP53 and APC mutation	C.3082+811TTTTAACA > GGTTTGAT (0.062) c.3082+96A > G (0.061) c.3082+99A > C (0.061) c.3082+1430C > T (0.094) c.3083–731TAAAAAAAAAAAT > TAAAAAAAAAAAAT (0.083) c.3083–730 del1 (0.073)
PS-14-549	F	62	Rectosigmoid colon	Colon cancer with distant metastasis	3+	165.18	No (1:1.1)	KRAS-wild BRAF V600E mutation Microsatellite-stable	c.3082+811TTTTAACA > GGTTTGAT(0.272) c.3082+63GT > TC (0.093) c.3082+69TATT > TAAGC (0.089) c.3082+74T > C (0.098) c.3082+376GAAGC > AGCCG (0.232) c.3082+730TGAGTCA > CAACATGA(0.109) c.3082+1061T > G (0.278) c.3082+1082GAAAAAAAAAC > GAAAAAAAAAAC (0.050) c.3083–1342AG > GT (0.062) c.3083–1101GGCC > TTAT (0.231) c.3083–1070CC > GT (0.159) c.3083–1063A > G (0.062) c.3083–731TAAAAAAAAAAAT > TAAAAAAAAAAT (0.081) c.3083–523TACC > AATT (0.051) c.3083–330CAATTG > GAAAAA (0.054) c.3083–324CT > TA (0.052) c.3083–208GGGTAAAA > ACAGGAAG (0.054)
PS-14-260	M	52	ACUP	Multiple lymph node enlargement without primary	3+	193.64	No (1:1.4)	KRAS-wild BRAF-wild TP53 and STK11 mutation Microsatellite-stable	N(Continued )ot tested
PS-14-503	M	67	Gastric	Gastric cancer with ascites	3+	□ 121	Yes (1:12.8)	TP53 p.V73M mutation	c.3082+811TTTTAACA > GGTTTGAT (0.134) c.3082+1082GAAAAAAAAAC > GAAAAAAAAAAC (0.055) c.3083–731TAAAAAAAAAAAT > TAAAAAAAAAAT (0.089)
PS-14-658	M	27	Gastric	Gastric cancer with multiple lymphadenopathy and ascites	3+	□ 131	No (1:0.75)	TP53 p.R141H mutation CDH1 p.G352fs mutation Polysomy-7	c.3082+811TTTTAACA > GGTTTGAT (0.070) c.3083–731TAAAAAAAAAAAT > TAAAAAAAAAAT (0.117) c.3083–730del1 (0.088)
PS-14-875	F	64	Gastric	Gastric cancer with seeding	3+	□ 142	No (1:1)	PTEN P.V119D mutation	c.3082+811TTTTAACA > GGTTTGAT (0.113) c.3082+730TGAG > CAACA (0.056) c.3082+735C > G (0.057) c.3082+1281GCAGAGCTT > TAAAAGGAG (0.062) c.3083–1320A > C (0.082) c.3083–1317G > T (0.082) c.3083–1041GT > CA (0.055) c.3083–860GTCAGTTGC > AACACTCAG (0.068) c.3083 731TAAAAAAAAAAAT > TAAAAAAAAAAT (0.113) c.3083–730del1 (0.075) c.3083–208GGGTAAAA > ACAGGAAG (0.087)

**Figure 2:**
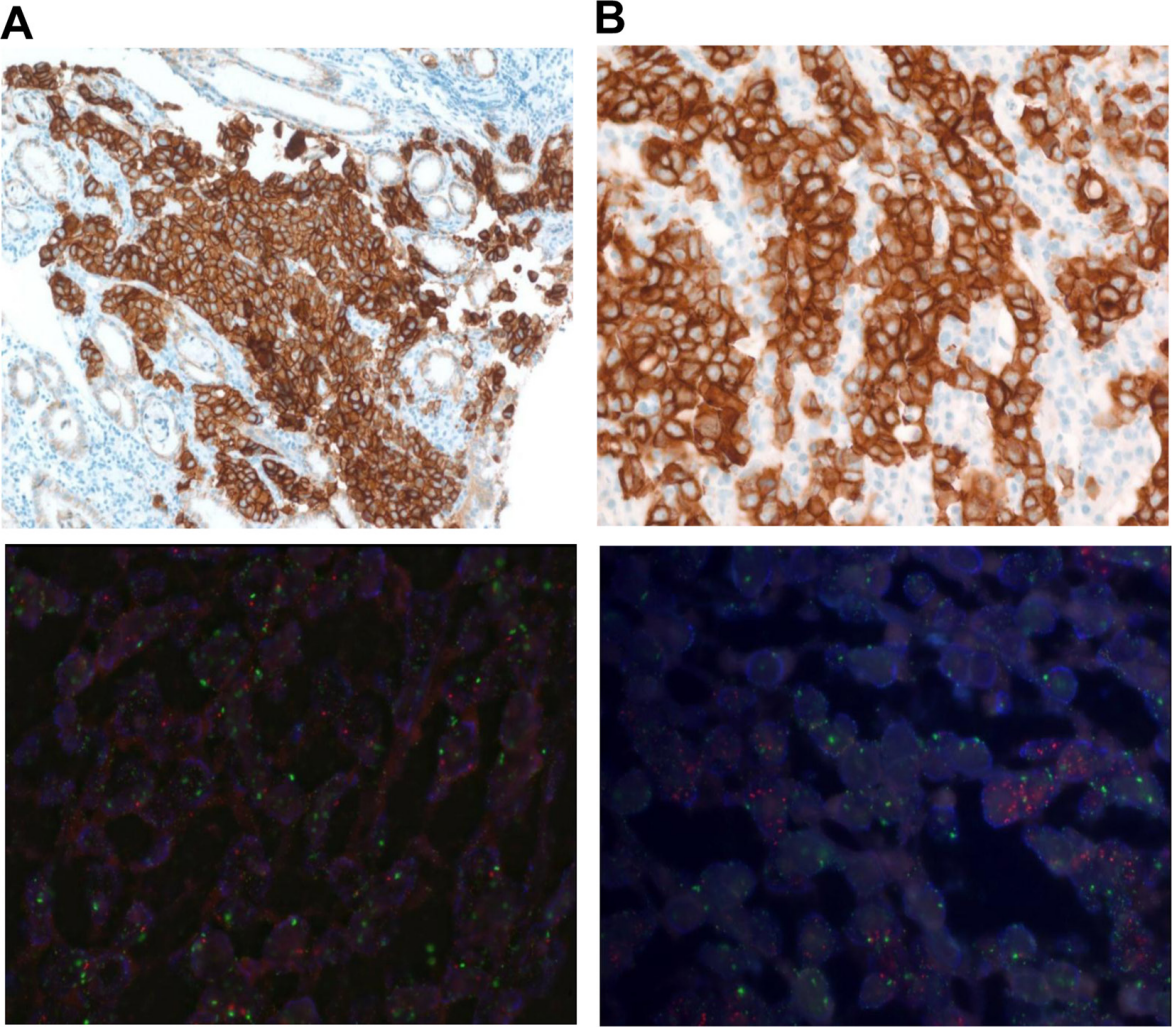
**A.** *METex14*+ GC with MET protein overexpression by IHC (upper panel) and no MET amplification by FISH (lower panel) **B.** *METex14*+ GC with MET protein overexpression by IHC (upper panel) and concomitant *MET* amplification by FISH (lower panel).

A total of 3 out of 42 GC patients were *METex14del* positive (Table 3). All GC cases were MET IHC 3+ and the only case in the series with *MET* amplification. For example, one case was a 27-year old male patient who presented with poorly differentiated adenocarcinoma and massive malignant ascites and died shortly after diagnosis. His tumor showed strong MET overexpression by IHC (3+) but no *MET* amplification by FISH (Figure 2a and 2b (with both amplification and *METex14*+), Table 3). PDC cell lines were generated from his malignant ascites and investigated for anti-tumor activity by MET inhibitors (below). The second *METex14del* case was a 67-year old male patient who also presented with poorly differentiated adenocarcinoma with concomitant *MET* amplification (MET/CEP7 ~12.8) and strong MET overexpression. For colon cancer, 4 patients were *METex14del* positive (Tables 2 and 3). All of the *METex14del*+ (or positive) colon cancer patients were not *MET* amplified and all but one were MET IHC 3+. *KRAS* was wild-type in all 4 colon cancer patients but BRAF V600E was detected in two of the 4 cases. Interestingly, all 4 colon cancer *METex14*+cases were left-sided colon cancer. For NSCLC, 5 of 51 (9.8%) patients were *METex14del*+ and none of the patients had concomitant *MET* amplifications. The median age for the five patients was 49 years and four patients (80%) were never-smokers (Table 2). Of the *METex14del*+ NSCLC patients, one patient had concomitant *EGFR* deletion mutation in exon 19 and T790M within exon 20. None of the *METex14del* NSCLC patients had concomitant *KRAS* mutations or *MET* amplification.

### METex14del patient derived tumor cell lines

**Figure 3:**
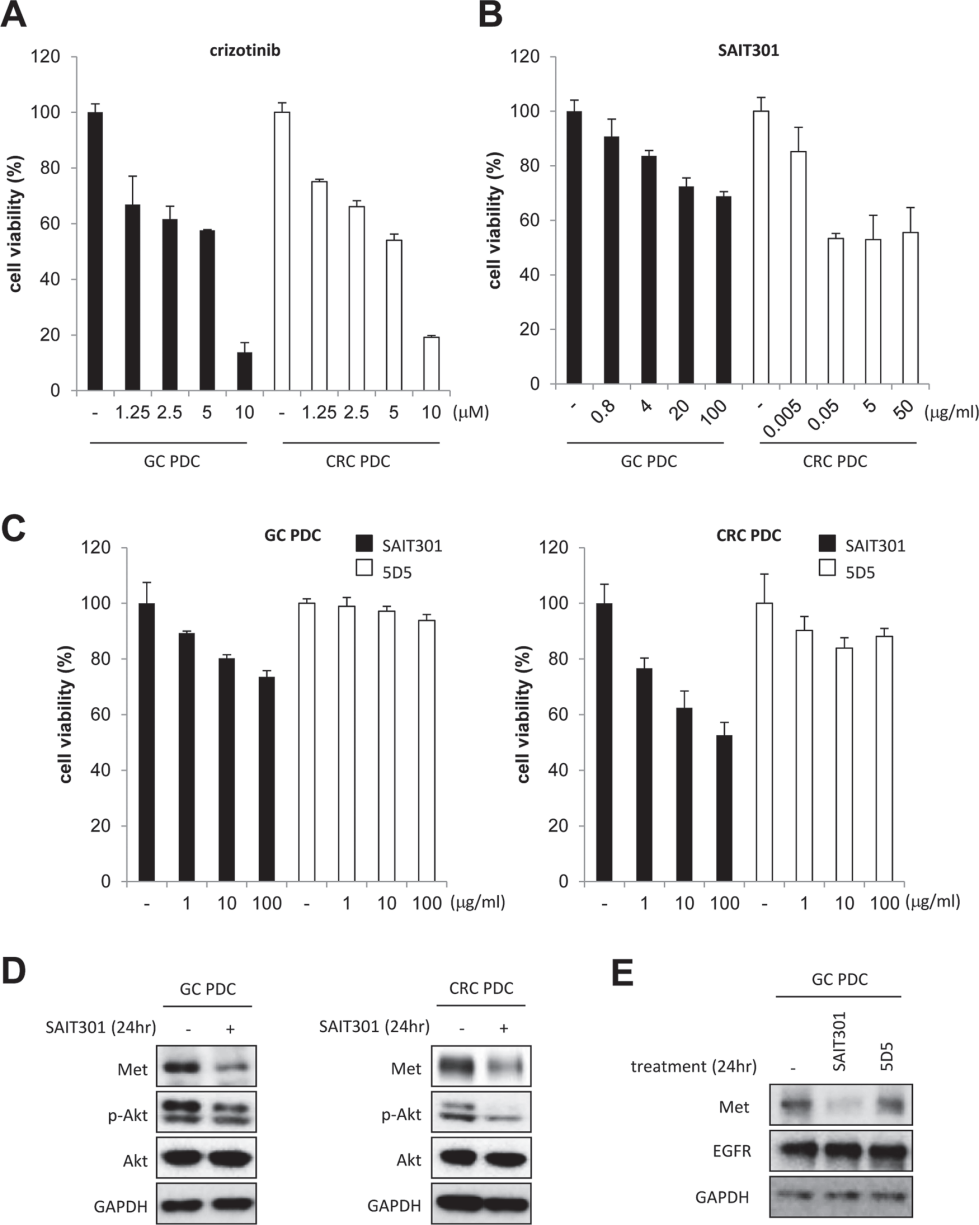
The anti-tumor efficacy of crizotinib and SAIT301 in *METex14+* GC and CRC PDCs **A.** and **B.** The viability of *METex14+* GC (■) and CRC (□) PDCs by CTG assay after treating with indicated concentrations of crizotinib (A) and SAIT301 (B) for 5 days. **C.** The viability of PDCs was measured by CTG assay after treatment with various concentrations of SAIT301 (■) and 5D5 (□) for 5 days. The relative cell viability (%) represents the percent growth as compared to the control group (no treatment). **D.** The protein levels of MET and p-Akt were measured by Western blot in GC and CRC PDCs after 24 h treatment of SAIT301. **E.** The MET protein levels were measured by Western blot in GC PDC after 24 h treatment of SAIT301 and 5D5.

We identified 5 with high *METex14* transcript expressions and further confirmed by RT-PCR (Table 1, Supplementary Figure S2). Of the 5 PDC cell lines, four *METex14del*+ cell lines were tested for potential anti-tumor efficacy of c-MET inhibitors, crizotinib (small molecule) and SAIT301 (monoclonal antibody) [6]. In Figure 3D, the expressions of MET protein in GC and CRC PDC cell lines were confirmed using Western blot analysis. Crizotinib, a small molecule targeting MET as well as ALK [12–14], led to dose-dependent growth inhibition both in GC and CRC PDCs (Figure 3A). We further tested otherMET inhibitors such as PHA-665752 and cabozantinib (XL184) (Supplementary Figure 3A and 3B); PHA-665752 is a small molecule inhibitor that specifically targets MET, and cabozantinib is a small molecule inhibitor that targets the MET, VEGFR2, and Ret kinases [15]. PHA-665752 and cabozantinib demonstrated potent growth inhibition in *METex14+* GC and CRC PDCs whereas lapatinib, an EGFR and HER2 inhibitor, and cetuximab (Erbitux), an EGFR targeting monoclonal antibody exhibited no such effects (Supplementary Figure S3C). Other PDC line data including melanoma and esophageal squamous cell carcinoma are provided in Supplementary Figure S4. Taken together, these experimental results implicate anti-MET drugs specific inhibition of *METex14del+* patient derived cell lines.

Previously reported anti-MET antibody, SAIT301 promotes a Cbl-independent MET degradation pathway and internalization of MET without ubiquitination [6]. Splice mutations of exon 14 have been associated with a deletion of the juxtamembrane domain of MET resulting in the loss of interaction with Cbl and Cbl-dependent MET degradation through ubiquitination. We tested SAIT301 in the *METex14del+* GC and CRC PDCs to further confirm its Cbl-independent Met degradation mechanism. As shown in Figure 3B and 3C, SAIT301 demonstrated potent growth inhibition of both GC and CRC PDCs whereas 5D5, another bivalent Met targeting antibody, exhibited no proliferation inhibitory effects. To determine antibody mediated down-regulation of MET, we measured the total MET levels in GC and CRC *METex14del*+ PDCs following SAIT301 treatment (Figure 3D). SAIT301 antibody dramatically reduced MET protein levels. In addition, phosphorylation of Akt, one of major signaling mediators of MET RTK, was also significantly inhibited by treatment with SAIT301. In conclusion, these results confirm that SAIT301 induces degradation of MET in *METex14*+ PDCs by down-regulating MET in Cbl-independent manners and subsequent inhibition of tumor cell growth.

## MATERIALS AND METHODS

### Patients

Patients with metastasis of solid cancers were enrolled onto the NEXT-1 trial [NCT#02141152] at Samsung Medical Center. The study was approved by the institutional review board of the Samsung Medical Center. All study participants provided written informed consent before study entry. Briefly, patients with metastatic solid cancer were eligible to enter the study. From November 2013 to August 2014, 428 patients were enrolled. of 428, 230 patients cohort with available tissue specimens for RNA extractions were included in this screening project.

### NanoString-Based multiplexed MET exon 14 deletion and ALK, ROS1, NTRK1, NTRK3 or RET fusion transcripts assay

We performed nCounter assays (NanoString, Seattle, WA) according to the manufacturer's instruction in duplicate. Total RNA was extracted from fresh tumor tissue (tumor content > 70%) or from one to four FFPE tissue sections (4 μm thick) using the High Pure RNA Paraffin kit (Roche Diagnostic, Mannheim, Germany). After RNA extraction according to the manufacturer's protocol, we added additional DNase treatment. RNA concentration was measured with the Nanodrop 8000 (Thermo-Scientific, Wilmington, DE) and stored −80°C until use. Briefly, 300 ng of total RNA was hybridized to nCounter probe sets for 16 hours at 65°C. The target sequence for *METex14del* transcript was 5′-ATTACTACTTGGGTTTTTCCTGTGG CTGAAAAAGAGAAAGCAAATTAAAGATCAGTTTC CTAATTCATCTCAGAACGGTTCATGCCGACAAGTG CAGTAT (Supplementary Figure S1A). For control, we used GAPDH exon 1–2 (accession number NM_002046.3), GUSB exon 4–5 (accession number NM_000181.1), OAZ1 (accession number NM_004152.2) and POLR2A (accession number NM_000937.2). Samples were processed using an automated nCounter Sample Prep Station (NanoString Technologies, Inc., Seattle, WA) as previously described [9]. Full probe sets are provided in Supplementary Table S1. Probe designs are described in previous work [16, 17].

### Validation of nanostring results by quantitative RT-PCR

RNAs were reverse transcribed using a superscript III first-strand synthesis system (Invitrogen, Carlsbad, CA). For validation of nanostring results, we designed forward primer for exon 13 of *MET*, GFPT1 F (5′-TGGGTTTTTCCTGTGGCTGAA-3′), reverse primer for exon 15 of *MET* (5′- GCATGAACCGTTCTGAG ATGAATT-3′) and probes overlapping an exon 13-exon 15 junction (5′- AAGCAAATTAAAGATCAGTTTCC-3′). GAPDH gene (ID; Hs99999905_m1) was used as an endogenous control. TaqMan probes were labelled with the reporter dye molecule FAM (6-carboxyfluorescein) at the 5′ end and with TaqMan minor groove binder non-fluorescent quencher (MGB-NFQ) probe at the 3 the 3in) at the 5′ end and with GATCAGTT TaqMan Universal PCR master mix with AmpErase UNG (Applied Biosystems), 900 nm primers (forward and reverse), 250 nm TaqMan probe, and 5 μl of cDNA sample in a total reaction volume of 20 μl. PCR conditions were 95°C for 10 min followed by 40 cycles of amplification at 95°C for 15 s and 60°C for 1 min on the ABI PRISM 7500HT Fast Real-time PCR. Ct values < 33 were considered as *METex14del*+ and ≥ 34 were negative for *METex14del*.

### Additional validation of nanostring results by CustomDx-Met001

To validate our results additionally, we used a qRT-PCR based kit for detection of alternatively spliced variant of MET to detect *METex14del*. The kit is intended to detect the presence of alternatively spliced (*METex14del*) MET transcript in RNA from FFPE tissue sections in accordance with the provided protocol (Custom Diagnostics, Irvine, CA). For the *MET* WT control, Ct from *MET* WT P/P mix should be in range between 22 and 28, and Ct from *METex14del* P/P mix should be “undetermined”. For the *METex14del* Control, Ct from *MET* WT P/P mix should be between 16 and 22; the Ct from *METex14del* P/P mix should be between 26 and 32. If the Ct values for controls fall outside the expected range then that run should not be used for evaluation of test samples.

### MET immunohistochemistry

For MET immunohistochemistry, we used CONFIRM anti-Total MET (SP44) rabbit monoclonal primary antibody (Ventana Medical Systems, Tucson, AZ, USA) with a Ventana BenchMark XT automated slide processing system according to the manufacturer's protocol as previously described [4, 18]. Both membranous and cytoplasmic staining was scored as follows: 0, no reactivity or faint staining; 1+, faint or weak staining; 2+, moderate staining; 3+, strong staining in > 10% of tumor cells. Membranous alone staining was scored by consensus recommendation on HER2 scoring for gastric carcinoma [19]: 0, no reactivity; 1+, faint/barely perceptible membranous reactivity; 2+, weak to moderate complete or basolateral membranous reactivity; 3+, moderate to strong complete or basolateral membranous reactivity in > 10% of tumor cells. MET overexpression was defined as 2+ or 3+ by membranous and cytoplasmic interpretation and only 3+ by membranous interpretation as previously described [4, 18].

### Fluorescent and bright-field double *in situ* hybridization

FISH was performed using dual-color DNA-specific *MET/*CEP7 probes (Abnova, Walnut, CA, USA) as described previously [11]. Two pathologists (S.A and M.H) counted the numbers of *MET* and chromosome 7 centromere probe (CEP7) signals (1 for individual signals, 6 for small clusters and 12 for big clusters) in 20 inter-phase tumor cell nuclei, and the mean number of *MET* and CEP7 copies per nucleus were determined, along with the ratio. Normal *MET*/CEP7 signals (one to two copies per cell) in the various non-neoplastic cells served as the internal positive control. We defined *MET* gene amplification as a *MET*/CEP7 ratio > 2.0 in 20 tumor nuclei and polysomy-7 were regarded as negative for gene amplification.

### Immunoblot analysis

Total proteins from PDCs were isolated using RIPA buffer (Sigma-Aldrich, St. Louis, MO, USA) containing a protease inhibitor cocktail (Roche, Mannheim, Germany) and phosphatase inhibitor cocktail (Roche), and protein concentrations were determined according to Bradford procedure using a Quick Start Bradford Protein Assay (Bio-Rad, Hercules, CA, USA). Thirty μg of proteins were subjected to 10% SDS-polyacrylamide gel electrophoresis, and electro-transferred onto nitrocellulose membranes. The membranes were blocked with 5% nonfat dry milk in Tris-buffered saline containing 0.1% v/v Tween 20, and probed overnight at 4°C with a Specific antibodies: pMET (Tyr 1234/1235), pAkt (Ser473), Akt(C67E7), pERK1/2 (Thr202/Tyr204), ERK1/2 (Thr202/Tyr204), GAPDH from Cell Signaling Technology (Beverly, MA, USA), and MET from Abcam (Abcam, Cambridge, UK) and MET (C-28) from Santa Cruz biotechnology (Santa Cruz, CA, USA), and beta actin from Sigma Aldrich. Horseradish peroxidase-conjugated anti-rabbit or mouse IgG (Vector, Burlingame, CA, USA) were used as a secondary antibody, and signals were detected by chemiluminescence using ECL Western Blotting Substrate (Thermo Scientific, Rockford, IL, USA), and visualized by using LAS-4000 (Fujifilm, Tokyo, Japan).

### Reagents

SAIT301 was produced using a recombinant CHO stable cell line [20]. Crizotinib, PHA-665752, XL-184 and lapatinib were purchased from Selleck Chemicals (Houston, TX, USA).

### Patient derived tumor cell culture and cell proliferation inhibition assay

Patient derived tumor cells (PDCs) were isolated from malignant effusions, surgical tissues or biopsies after obtaining informed consent form (the SMC Oncology Biomarker study (NCT#01831609, clinicaltrials.gov). The protocol was approved by the Institutional Review Board at Samsung Medical Center. The cells were cultured in RPMI media supplemented with 10% fetal bovine serum, 0.5 μg/ ml of hydrocortisone (Sigma Aldrich), 5 μg/ ml of insulin(PeproTech, Rocky Hill, NJ, USA), 5 ng of EGF and FGF (PeproTech). Cell proliferation in response to antibody treatment *in vitro* was assessed by a CTG (Promega, Madison, WI, USA) assay according to manufacturer instructions. Cells were plated at a density of 5 × 10^5^ cells in FBS 10% (v/v) RPMI 1640 medium onto a 96-well plate (BD Biosciences, Palo Alto, CA, USA). After 24 h incubation, treated antibodies or small molecules diluted in 10% FBS (v/v) RPMI medium were added. After 5 days incubation, 100 μl of the CTG reagent was added to each well followed by incubation at RT for 30 min. The luminescence signal was recorded using Envision 2104 Multi-label Reader (Perkin Elmer, Foster City, CA, USA).

## DISCUSSION

The Met proto-oncogene is encoded by 21 exons spanned by 20 introns [7]. The transmembrane domain of MET is encoded by the whole of exon 13 and part of exon 14. Met exon 14 deletion thus results in an in frame deletion of 47 amino acids in the juxtamembrane region which contains the domain. The *MET* deletion mutant, while displaying decreased Cbl binding, leads to prolonged protein stability, extended cell signaling on ligand stimulation, and increased tumorigenicity [9]. The incidence of *METex14del* was estimated to be 3.5% in NSCLC [5] and 1.9% in neuroblastoma [11]. Recently, analysis of tumor genomic profiles from 38,028 patients identified 221 cases with *METex14* mutations (0.6%), including 126 distinct sequence variants in lung adenocarcinoma (3.0%), other lung neoplasms (2.3%), brain glioma (0.4%), and tumors of unknown primary origin (0.4%) [21]. To date, *METex14del* has not been reported in either gastric or colon cancer patients. This is the first report that identified *METex14del* at a frequency of approximately 5% in both gastric and colon cancer in addition to NSCLC. All *METex14del*+ cases also over-expressed MET protein with only one case showed *MET* amplification consistent with the hypothesis that *METex14del* leads to MET over-expression without the need for concurrent *MET* amplification. In addition, *METex14del* occurs exclusively to ALK, ROS1, NTRK1, NTRK3 or RET fusions indicating METex14del is likely a driver mutation and defines a unique molecular subset of gastric and colon cancers.

In GC, only Hs746T cell line exhibited both splice-site mutations and *MET* amplification with MET protein overexpression [9]. We are the first group to report on three GC cases (4.8%) with *METex14del* and strong MET protein overexpression by IHC. We performed *MET* exon 14 Sanger sequencing with gDNAs and cDNAs, but failed to detect mutations in our GI *METex14del*+cancer samples (*data not shown*). Given the low sensitivity of Sanger sequencing (<12%) and low Ct values in our RT-PCR results, we postulate that *METex14del*+ tumor population is present in small subpopulation of tumors. Furthermore, we also report c.3082+811A TTTTAACA > GGTTTGAT mutations on intron 14 region of *MET* with variant allele frequencies around 10%. This intronic mutation is a novel mutation, which has not been reported in COSMIC and TCGA lung adenocarcinomas (Supplementary Table S2). We found that this mutation site is important where proteins including well known splicing factors such as Jun, and Fox, etc. bind. ChIP-Seq dataset of ENCODE project provides strong evidences and the specific intronic mutation site reported here exists in the middle of the protein binding sites (Supplementary Figure S5). We assume that this 8bp mutation on this binding site would affect decreased protein binding affinity of these proteins and may cause exon 14 skipping in small subpopulations of tumor, especially given the tumor heterogeneity in GI cancer.

In two PDC cell lines with *METex14del*+ without concurrent *MET* amplification, tumor growths were profoundly inhibited by both MET tyrosine kinase inhibitors and a MET targeting monoclonal antibody-. Our study is the first proving efficacy of MET inhibitors or monoclonal antibody in human GI PDC lines with *METex14del* that over-expressed MET but did not have *MET* amplification. Furthermore the *in vitro* cell line inhibition data indicated that *METex14del* is potentially an actionable driver mutation in GI malignancies. This finding provides new opportunities for clinical trials on MET inhibitors in metastatic GC to include not only *MET* amplified GC, but also MET over-expressed, *MET* non-amplified and *METex14del+* GC. So far all *METex14del*+ cases had concurrent MET over-expression, it remained to be determined if *METex14del*. Now, we developed screening algorithm to detect *METex14del* for screening oncology patients. First, we screen MET overexpression by IHC and select MET-positive (≥ 2+) cases.^4^ In IHC-positive cases, we perform FISH to exclude *MET* amplification as a cause of MET overexpression. In cases without *MET* amplification, we perform custom-designed and RT-PCR using mRNAs from tumor to detect *METex14del* transcripts. For RT-PCR positive cases, we sequence them to find underlying cause of *METex14* alterations at the DNA level.

In summary this report supports that the aberration in Cbl-mediated negative regulation of MET can indeed result in MET protein overexpression and subsequent addiction of tumor cells to MET signaling and may serve as an actionable driver mutation in a subset of GI malignancies.

## SUPPLEMENTARY FIGURES AND TABLES






