# Supplementary material for: Gastrointestinal malignancies harbor actionable MET exon 14 deletions
Source: Oncotarget. 2015 Sep 10;6(29):28211–22. doi: 10.18632/oncotarget.4721 (PMC4695055; doi:10.18632/oncotarget.4721)
Supplement: Supplementary file 1 [file oncotarget-06-28211-s001.pdf]

SUPPLEMENTARY FIGURES AND TABLES

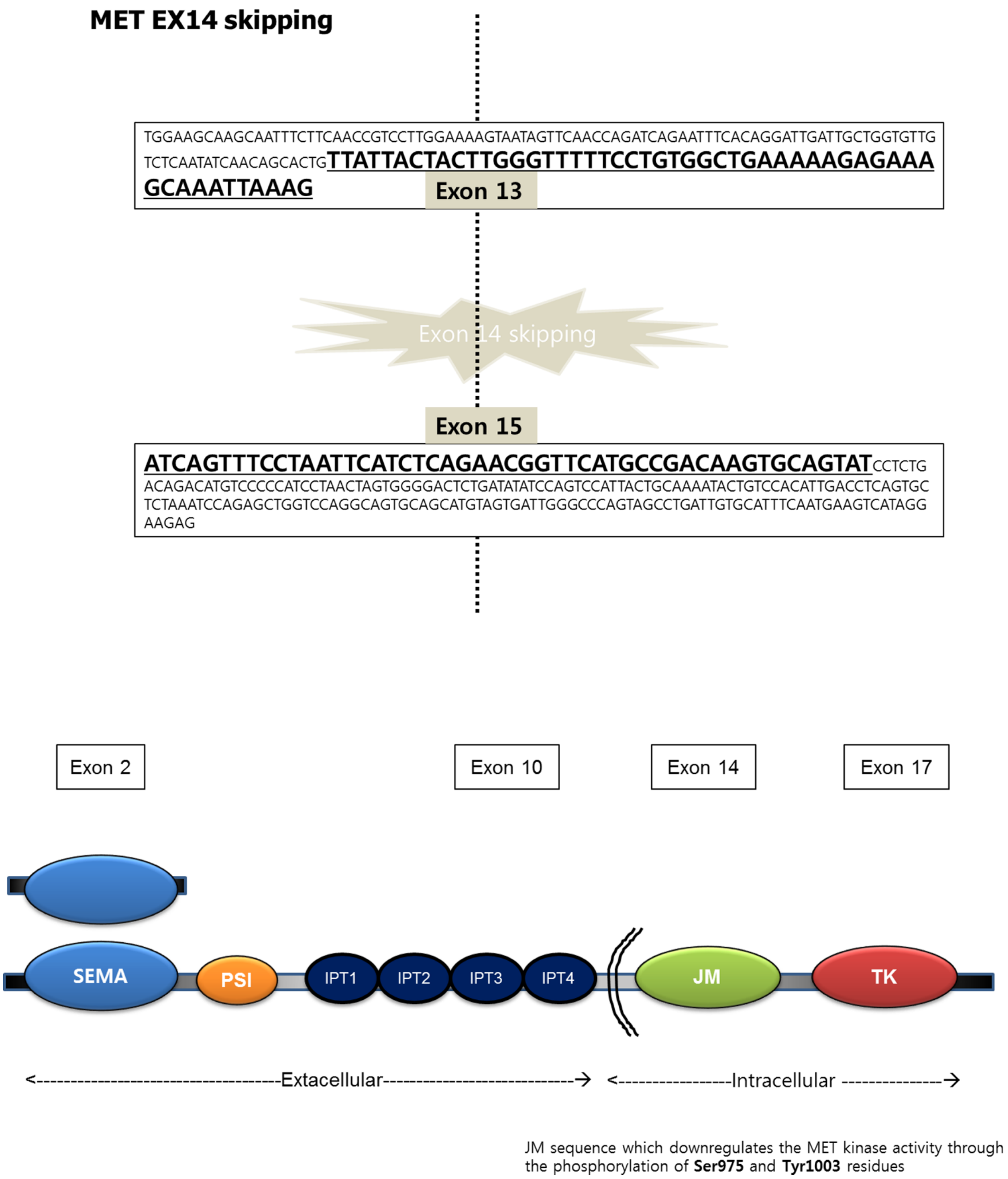

Supplementary Figure S1: Structure of MET.

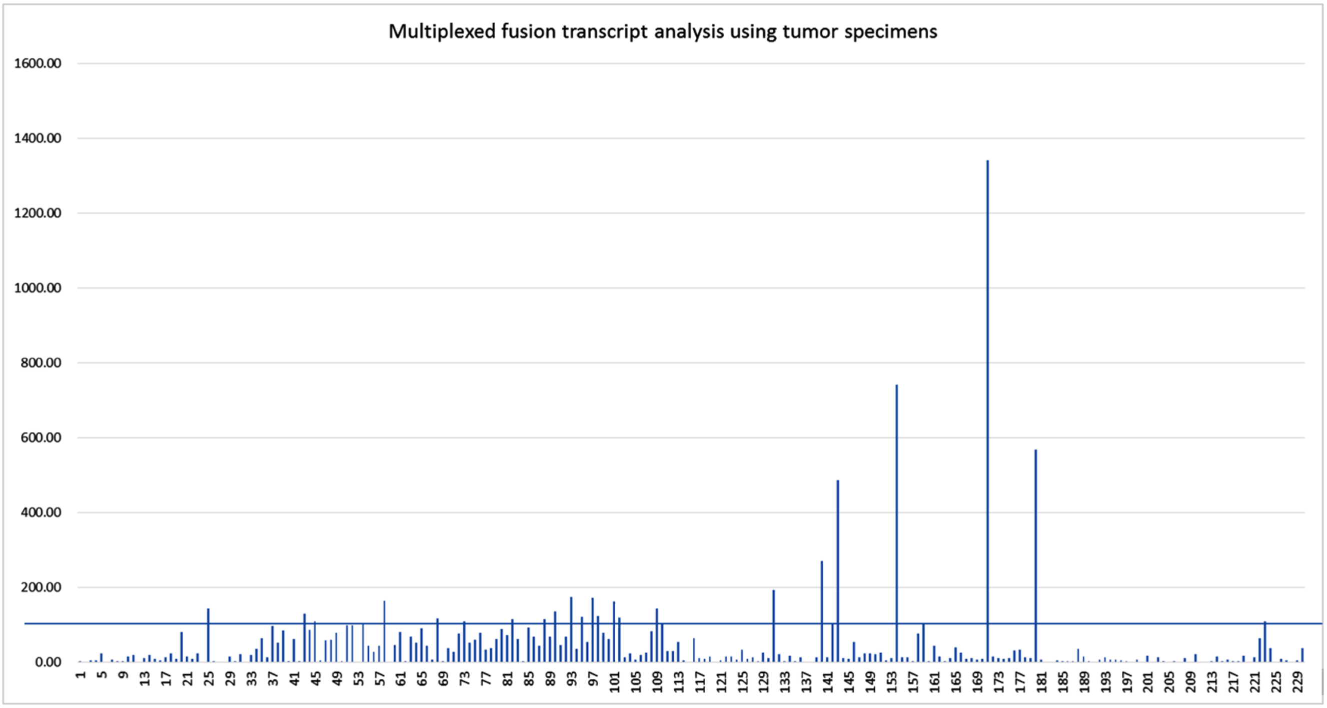

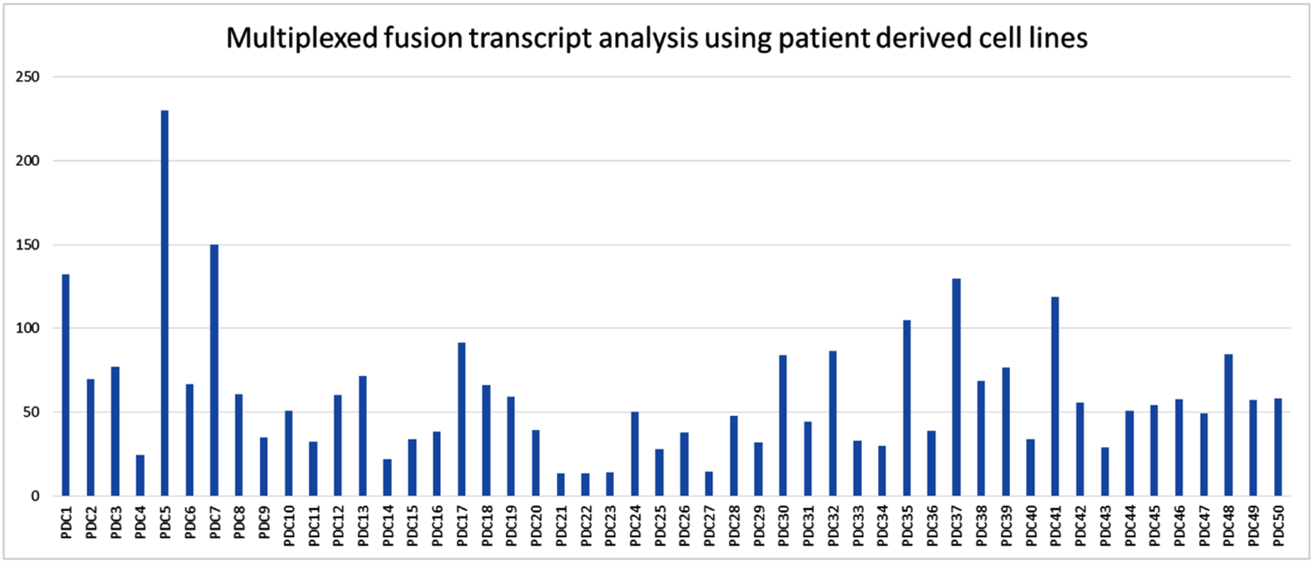

Supplementary Figure S2: Multiplxed fusion transcript analysis using nCounter assay.

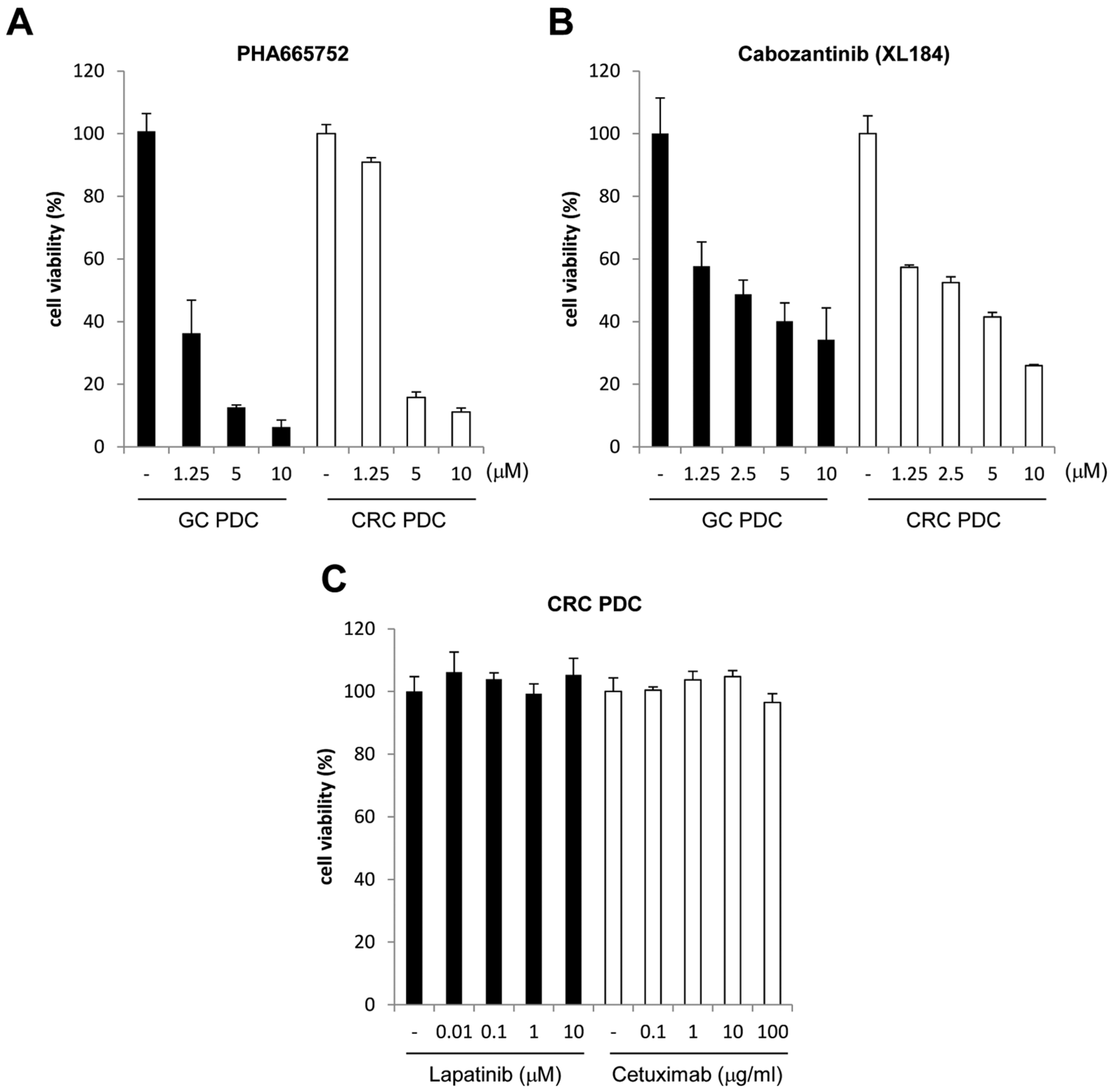

Supplementary Figure S3: The anti-tumor efficacy of PHA665752, cabozantinib and anti-EGFR targeted drug in METex14 GC & CRC PDCs.

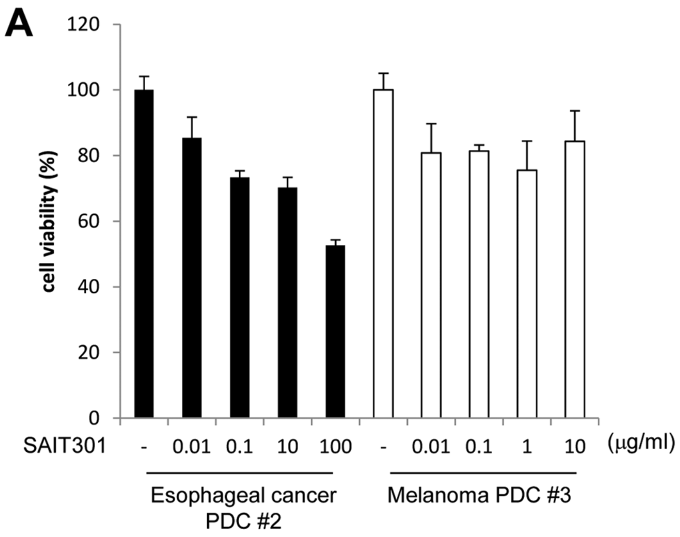

Supplementary Figure S4: The anti-tumor efficacy of anti-MET treatment in esophageal cancer, melanoma PDCs.

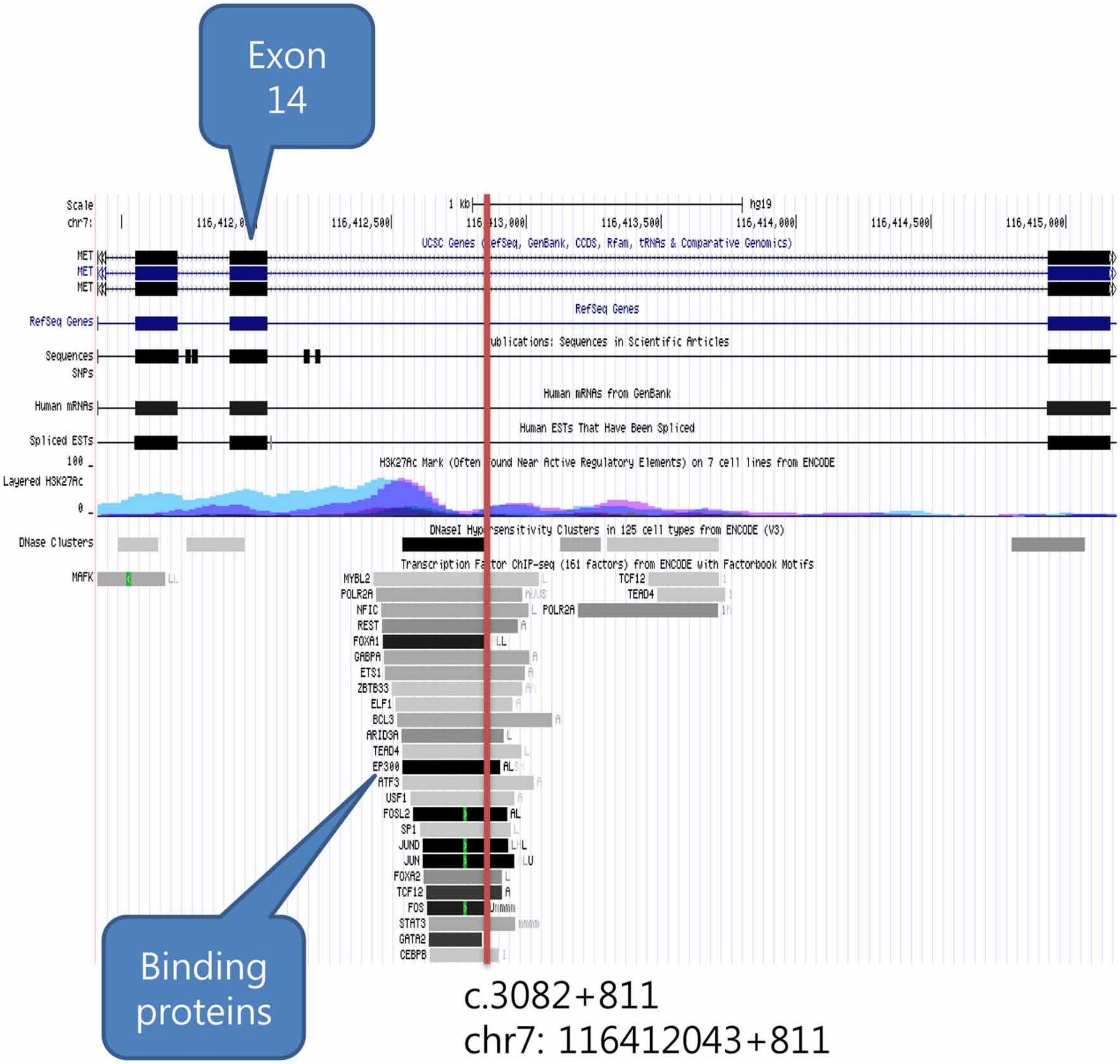

Supplementary Figure S5: Exon 14 and flanking intron of MET gene with c.3082+811 site (red bar) in chromosome 7.

Supplementary Table S1: Nanostring probe design.

Supplementary Table S2: METex14 in COSMIC and TCGA lung adenocarcinomas data.
